# Depth-Dependent Cellular Response from Dental Bulk-Fill Resins in Human Dental Pulp Stem Cells

**DOI:** 10.1155/2019/1251536

**Published:** 2019-10-24

**Authors:** Su-Min Lee, Soo-Youn Kim, Jae-Heon Kim, Soo-Kyung Jun, Hae-Won Kim, Jung-Hwan Lee, Hae-Hyoung Lee

**Affiliations:** ^1^Department of Biomaterials Science, College of Dentistry, Dankook University, Chungnam, Cheonan 31116, Republic of Korea; ^2^Department of Dental Hygiene, Kyungdong University, Wonju 26495, Republic of Korea; ^3^Institute of Tissue Regeneration Engineering (ITREN), Dankook University, Cheonan 31116, Republic of Korea; ^4^Department of Nanobiomedical Science & BK21 PLUS NBM Global Research Center for Regenerative Medicine, Dankook University, Cheonan 31116, Republic of Korea; ^5^UCL Eastman-Korea Dental Medicine Innovation Centre, Dankook University, Cheonan 31116, Republic of Korea

## Abstract

The proper choice of dental composite resins is necessary based on the minimal cytotoxicity and antiodontogenesis on human dental pulp stem cells for dental pulp-dentin tissue repair and regeneration. The aim of this study was to evaluate the cytotoxicity and antidifferentiation effects of dental bulk-fill resins, able to be polymerized as a bulk status for filling deep cavity of a tooth by single light curing, against human dental pulp stem cells (hDPSCs) from three compartments corresponding to depth (0-2, 2-4, and 4-6 mm) from the light-curing site. Three bulk-fill composite resins (SDR, Venus bulk-fill (VBF), and Beautifil Bulk Flowable (BBF)) and a conventional flowable composite resin (Filtek Z350 XT flowable restorative (ZFF)) were individually filled into a cylindrical hole (*h* = 2 mm, *Ф* = 10 mm), and three compartments (total ~6 mm of height) were combined as a single assembly for light curing. The resin samples from the three layers were separated and eluted in the culture medium. The extracts were exposed to hDPSCs, and cytotoxicity and differentiation capability were evaluated. Depth of cure and surface hardness according to depth were determined. All bulk-fill resins except BBF revealed cytotoxicity from 4 to 6 or 2 to 4 mm, while ZFF was cytotoxic at over 2 mm. Depth of cure was detected from 3.55 to 4.02 mm in the bulk-fill resins (vs. ~2.25 mm in conventional resin), and 80% hardness compared with that of a fully polymerized top surface was determined from 4.2 to 6 mm in the bulk-fill resin (vs. 2.4 mm in conventional resin). Antidifferentiation was revealed at a depth of 4-6 mm in the bulk-fill resin. There was a difference in depth of cytotoxicity and antidifferentiation between the bulk-fill composite resins, which was mainly due to different cure depths and ingredients. Therefore, careful consideration of choice of bulk-fill resins is necessary especially for restoration of deep cavities for maintaining the viability and differentiation ability of dental pulp stem cells.

## 1. Introduction

Teeth are unique and complex organ, containing both soft tissue (pulp) and hard tissue (dentin and enamel), because teeth are ectomesenchymal origin including epithelial cells (ectoderm) and cranial neural crest-derived mesenchymal cells (mesenchyme) [1, 2]. Particularly, dental pulp tissue is very important to ensure the viability or to repair/regenerate tooth complex, and it contains blood vessels, nerves, connective tissue, and stem cell niches [3]. Among them, dental pulp stem cells are highlighted as the key component for repair/regeneration of teeth, capable of regenerating most part of dental pulp tissue in animal and human models as postnatal stem cells [4–6]. Occasionally, dental pulp stem cells are damaged before, during, or after dental practice due to bacterial infection (mostly from dental caries), iatrogenic factors (heat or mechanical force), or cytotoxic components from dental materials deposited above the pulp tissue for dental cavity restoration [7]. Thus, any adverse effects of viability and odontogenesis, ability to differentiate dental pulp stem cells for pulp tissue repair/regeneration, have been carefully investigated by dental scientist during the development and usage of dental restorative materials [8, 9].

Composite resins are popular restorative materials in dentistry due to their adequate strength, characteristics of adhering to teeth, and optical properties [10, 11]. They resemble tooth colour and are available in different shades, which gives them an advantage in aesthetics [12, 13]. However, they still have several drawbacks; specifically, composite resins shrink during polymerization, and problems such as increased sensitivity and microleakage can occur due to the gaps generated between the teeth and the material [14, 15]. Moreover, the depth of cure of conventional composite resins is limited to 2 mm; thus, incremental techniques are recommended in the filling [16]. The incremental placement requires long restoration times, and concerns of air inflow and contamination between the layers exist [17]. Additionally, conventional resins are difficult to apply in deep cavities due to limited depth of cure [18].

To tackle above drawbacks, bulk-fill composite resins were recently developed [19]. These new composites can be cured by a single light curing after bulk placement at depths up to 4~6 mm due to enhanced light penetration and low polymerization shrinkage. Based on preclinical studies that assessed the biological and physiomechanical performance of bulk-fill resins, they have been utilized to restore the enamel-dentin complex quickly and safely [17, 20, 21]. Studies assessing the clinical performance of bulk-fill resins in posterior teeth also revealed no differences in the failure rate between conventional and bulk-fill base/flowable resins [22].

However, there are still concerns regarding the cytotoxicity of bulk-fill resins, especially the lower parts, as light for polymerization may not penetrate deep enough and insufficient polymerization can occur [17]. Toh et al. reported that some eluted bulk-fill materials were cytotoxic to mouse fibroblasts, and extracts from specimens at a 4 mm depth showed more severe cytotoxicity than those from specimens at a 2 mm depth [23]. Other investigations determined the cytotoxicity to specific cell types in pulp tissue (dental pulp stem cells or cortical neuron) and yielded controversial cytotoxicity results depending on the cell types and other experimental details, such as methods of coculture (direct or indirect methods) and bulk-fill resin depths [17, 24].

Therefore, this study is aimed at evaluating the cytotoxicity against human dental pulp stem cells, which uncured resin monomers from bulk-fill composite resins may adversely affect through dentinal tubules, using (serially diluted) elutes obtained from different depth compartments (0-2, 2-4, and 4-6 mm) after single light polymerization. These depths match the probable thicknesses of bulk-fill resins in clinical settings, from the occlusal surface of the enamel to the roof of the pulp chamber (~6 mm). The null hypothesis was that there was no difference in the cytotoxicity of resin compartments according to the depth from the light-curing site.

## 2. Materials and Methods

### 2.1. Sample Preparation

**Figure 1:**
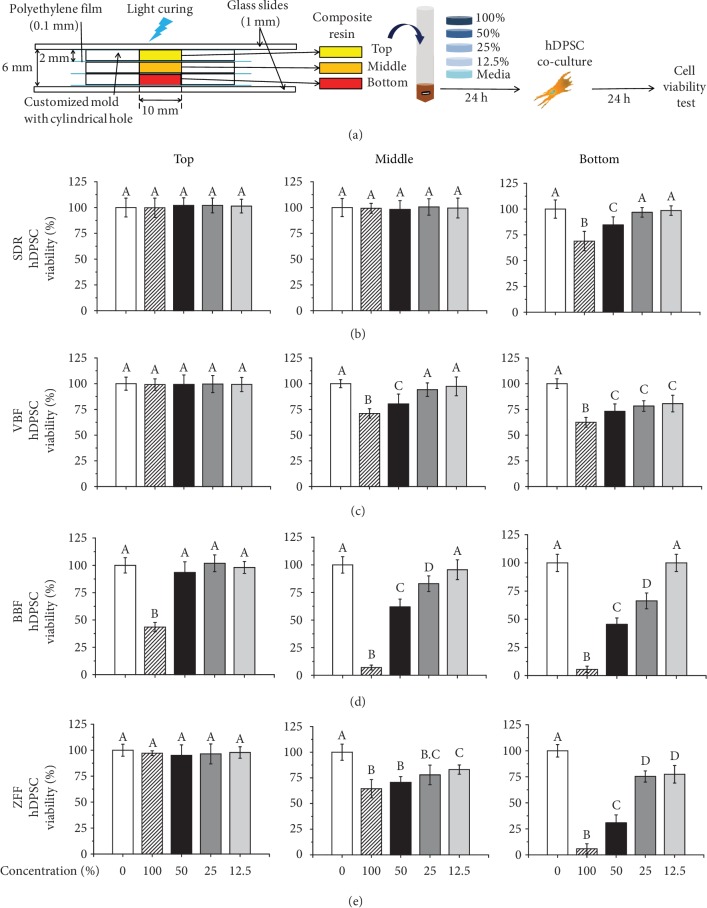
Schematic of the cytotoxicity test procedure with different depths of specimens from the light curing and results of cell viability. (a) Specimen preparation depending on the depth from the light and their extraction for the cytotoxicity test against human dental pulp stem cells (hDPSCs). (b-e) The results of the WST cell viability assay, which was dependent on the product (SDR, VBF, BBF (bulk-fill resins), and ZFF (conventional flowable resin)) and specimen depth (top (0-2 mm), middle (2-4 mm), and bottom (4-6 mm)), are shown in (b-e). The bottom samples showed the most cytotoxicity among the three compartments (top, middle, and bottom) in all groups. Compared with SDR and VBF, BBF and ZFF showed more cytotoxicity. Different letters indicate significant differences between groups (*n* = 6, *p* < 0.05).

Three bulk-fill composite resins, SDR, Venus bulk-fill (VBF), and Beautifil Bulk Flowable (BBF), and a conventional composite resin (Filtek Z350 XT flowable restorative (ZFF)) were used in the study (Table 1). The Teflon moulds were customized with cylindrical holes of 10 mm in diameter and 2 mm in thickness. The depths of 2, 4, and 6 mm were obtained by piling up the three moulds and placing polyethylene film between the layers. The polyethylene film was also placed beneath the bottom layer of the mould (Figure 1(a)). Composite resins were poured into the cavities of each mould in single increments, and the excess was extruded by compressing with a glass slide. The uppermost layer was covered with a 1 mm thick glass slide to flatten the surface and mimic clinical polymerization circumstance in the oral cavity (~1 mm apart from the top surface of resin). The samples were cured for 20 s using LED, with an irradiance of 1000 mW/cm^2^, which was checked before every experimental time point by an optical power meter (Digirate LM-100, Monitex, New Taipei City, Taiwan). The tip of the light was placed on the glass slide, which was illuminated vertically. Light curing was performed four times by moving the tip around in a circle, with as much area overlap as possible to evenly cover the entire 10 mm diameter. Next, the excess materials beyond the mould were removed, and the cured composite sample discs were separated from the mould for extraction.

### 2.2. Collection of Extracts

The sample discs were subsequently put in the culture media, which consisted of *α*-MEM mixed with 10% foetal bovine serum (Gibco), 1% penicillin/streptomycin (Invitrogen, Carlsbad, CA, USA), 1% GlutaMAX (Gibco), and 0.1% ascorbic acid (Sigma-Aldrich, St. Louis, MO, USA), which was used as the extractant. The volume of the extractant was determined according to the International Standards Organization (ISO) 10993-12. The preferred ratio of a sample surface area to extractant volume was 3 cm^2^/mL. The total surface area of one specimen was 2.2 cm^2^; thus, 0.73 mL of supplemented *α*-MEM was needed for each specimen. The four types of composite resin discs were completely immersed in the extraction media and incubated in the shaking incubator at 37°C for 24 h. Supplemented medium was also incubated. A shaking incubator (120 rpm) was used to mimic the clinically alterable oral environment.

### 2.3. Human Dental Pulp Stem Cell Culture

The hDPSCs were extracted from human third molars after the approval of the Institutional Review Board of Dankook University Dental Hospital (IRB number H-1407/009/004). Cells from low passages (under 10) were used. Pulp tissues were gathered antiseptically and put into phosphate-buffered solution (PBS) (Gibco, Grand Island, NY, USA) with 1% penicillin/streptomycin (Gibco). We added 0.08% collagenase type I (Worthington Biochemical, Lakewood, NJ, USA) for enzymatic digestion, which was followed by incubation for 30 minutes. Tapping was performed every 10 minutes. hDPSCs were then centrifuged at 1500 rpm for 3 minutes. Thereafter, the cells were supplied with *α*-MEM with 10% foetal bovine serum (Gibco), 1% penicillin/streptomycin (Invitrogen, Carlsbad, CA, USA), 1% GlutaMAX (Gibco), and 0.1% ascorbic acid (Sigma-Aldrich) and cultured in a humidified atmosphere of 5% CO_2_ at 37°C. All culture systems adhered to the above conditions.

### 2.4. Extract Test

The hDPSCs were gathered according to the previous protocol and seeded at a density of 1 × 10^5^ cells/mL in each well of a 96-well plate (SPL Life Sciences, Pocheon, Gyeonggi-do, Korea) with 100 *μ*L of supplemented *α*-MEM in a humidified atmosphere of 5% CO_2_ at 37°C for 24 h [25]. The 24 h incubated extracts and the supplemented medium (see Collection of Extracts) were filtered using 0.20 *μ*m filters (Corning, NY 14831, made in Germany) and syringes. Then, the plating media containing hDPSCs were washed with PBS (100 *μ*L), and the cells were cocultured with filtered eluates in 37°C for another 24 h. The filtrates were serially diluted with the previously incubated supplemented medium. The percentages of the final concentrations of extracts in the culture media were 100, 50, 25, 12.5, and 0% (the control group).

### 2.5. Evaluation of Cell Viability

The solutions of hDPSCs incubated in the eluates for 24 h (refer to Extract Test) were removed and washed with PBS. EZ-CYTOX (Daeillab Service, Guro, Seoul, Korea) was added to the supplemented *α*-MEM at 10% volume of the medium. EZ-CYTOX includes water-soluble tetrazolium salt (WST), which is reduced by dehydrogenase present only in the electron transport systems of the mitochondria of viable cells. Consequently, the orange-coloured substance formazan is produced (WST assay). Then, 100 *μ*L of the mixture was put in each cell-containing well, as well as in several blank wells. Following incubation in humid conditions of 5% CO_2_ at 37°C, optical absorbance was measured at a wavelength of 450 nm with a Multidetection Microplate Reader (Spectramax M2e, Molecular Devices, Sunnyvale, CA, USA) 2 h after injection of the dye. Higher absorbance indicated greater cell viability. Furthermore, to confirm the numerical cell viability, images of live and dead cells were obtained by a semiconfocal laser scanning microscope (Celena, Logos Biosystems, Anyang, Korea). After removing the media and washing with PBS, 0.5 *μ*M calcein AM and 2 *μ*M ethidium homodimer-1 solutions were added to the cells. Then, the cells were incubated at room temperature for 30 minutes. A cytotoxicity test was performed in sextuplicate (*n* = 6) in each group, and more than three independent experiments were carried out.

### 2.6. Measuring the Depth of Cure

Additionally, the depth of cure for each material was measured according to the ISO 4049 scraping test and Vickers hardness profile methodology [26, 27]. For the scraping test, two stainless steel moulds with cylindrical holes (*ϕ* = 4 mm and *h* = 6 mm) were piled up to provide a height of 12 mm, which is longer than twice the assumed depth of cure for bulk-fill composite resins. Moulds were stacked onto the polyethylene film. Four types of composite resins were poured into the holes of the moulds. Filled materials were covered with polyethylene film and pressed with a glass slide (*h* = 1 mm) to remove the excess. Then, LED LCU (VALO^TM^, Ultradent, USA) was cured from the top surface with an irradiance of 1000 mW/cm^2^ for 20 s. The cured specimens were separated from the mould, and the uncured soft parts of the composite resins were cut out with a plastic spatula. Then, the length of the left parts of the resins was measured. The depth of cure was determined by dividing the length by two (*n* = 5). After each resin specimen (*ϕ* = 10 mm and *h* = 6 mm) designated for extraction was light-polymerized by the aforementioned methodology without polyethylene film in between, Vickers hardness (HM-221, Mitutoyo, Tokyo, Japan) was measured at 500 gf (4.90 N) for 20 s on a cross-sectioned plain polished with up to 4000 grit SiC paper at every 0.5 mm increment (*n* = 3, measurement) from the top of the surfaces (0, 0.5, 1, 1.5, 2, 2.5, 3, 3.5, 4, 4.5, 5, 5.5, and 6 mm) to the end of the three different specimen [28]. A total of 9 values from each group were recorded.

### 2.7. Odontogenesis of hDPSCs with Elute

hDPSCs (1 × 10^5^ cells/mL) seeded in 24-well plates were cocultured with 12.5% elute for 7 days, with media change every 2-3 days. Elute from each specimen was gathered in odontogenic media further supplemented with ascorbic acid (50 *μ*g/mL), b-glycerophosphate (10 mM), and dexamethasone (100 nM) for differentiation, in addition to the above growth media, by the same extraction manner discussed above. Original elute (100%) was further diluted to the proper amounts with odontogenic media. To investigate odontogenic capacity, alkaline phosphate staining was performed. Five replicate samples were tested for each condition. Cultured cells were washed with PBS, and 200 *μ*L of FAST BCIP/NBT (B5655, Sigma-Aldrich) diluted into 10 mL of DW was added. After 1 h, alkaline-stained images were obtained by a microscope. The ALP-stained area was quantified by ImageJ (1.52e, NIH, USA) and normalized to the intensity obtained from the differentiation media control.

### 2.8. Statistical Analysis

The cytotoxicity data from different extraction starting points were statistically analysed by repeated measures analysis of variance (ANOVA) after performing the Shapiro-Wilk test to confirm normality. ANOVA was used for cytotoxicity comparison between serially diluted extract groups (100, 55, 25, 12.5, and 0%) within the same product and extract starting point. The Tukey post hoc test was used at levels of significance of *p* < 0.05. The SPSS PASW version 23.0 software program (SPSS Inc.) was used.

## 3. Results

### 3.1. Cytotoxicity against hDPSCs

The results of the WST cell viability assay are shown in Figures 1(b)–1(e). The viability of hDPSCs when immersed in the eluates from the top, middle, and bottom specimens of four different composite resins was measured. Overall, after incubation with extracts from the top specimen, which represented a 2 mm distance from the light-curing site, all groups except the undiluted 100% BBF showed ~100% cell viability similar to control (Figures 1(b)–1(e), *p* > 0.05). Only 43.49% of the cells survived in the undiluted extract of the top layer of BBF (Figure 1(d), *p* < 0.05). For the middle levels, there were no cytotoxic effects (~100% cell viability) at any of the concentrations of SDR elutes compared to control (Figure 1(a), *p* > 0.05), whereas cell viability gradually increased after serial dilution in other materials. In detail, in the middle layer, SDR showed ~100%, revealing no cytotoxicity at 100% concentration compared to control (Figure 1(a), *p* > 0.05). VBF and BBF yielded statistically different values (71.05% and 64.43%, respectively) of cell viability at 100% concentration compared to control (Table 2, *p* < 0.05) but did not show statistically different cell viability compared to control at 25% and 12.5% concentrations, respectively (~100%, Table 2, *p* > 0.05). However, the conventional flowable resin, ZFF, was still cytotoxic at 12.5% to some extent (~80%, *p* < 0.05). The bottom samples generally revealed the lowest cell viability among each concentration of three compartments (top, middle, and bottom) in all groups. The viabilities associated with SDR and BBF were ~69% and ~6% at 100% concentration (Table 2, *p* < 0.05), and these resins did not show statistically different cell viability compared to control at concentrations of 25% and 12.5% (~100%, Table 2, *p* > 0.05), respectively. In contrast, VBF and ZFF did not reach noncytotoxic levels (~100%) over continuous dilution to 12.5%. According to the above results, the null hypothesis that there was no difference in cytotoxicity of the resins depending on the distance from the light-curing site was rejected.

**Figure 2:**
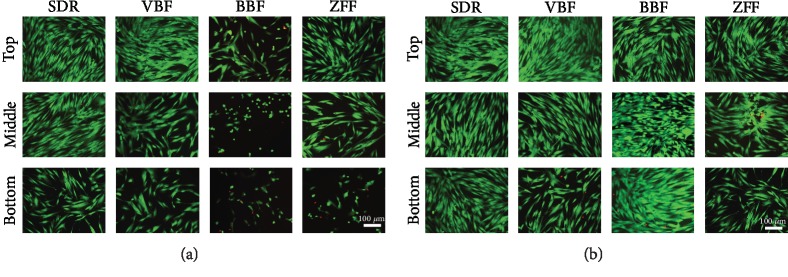
Live and dead staining of human dental pulp cells (hDPSCs) incubated with (a) 100% or (b) 12.5% elute from different specimen depths for 24 h. Live/dead cells were stained green/red, respectively, and representative images were shown (*n* = 6). Bottom specimens showed fewer live cells in all groups for 100% elute. BBF and ZFF yielded more live cells than SDR and VBF at 100% elute. The number of live cells generally increased from 100% to 12.5% concentrations. Representative data are shown after triplicate experiments.

Cytotoxicity from 100% cultured conditions was confirmed by live and dead cell staining using a semiconfocal microscope (Figure 2(a)). Live cells are indicated in green, and dead cells appear red in the images. At 100% concentrations of SDR, VBF, and ZFF, the bottom group showed 5~60% live cell numbers compared to the top group. Another bulk-fill resin, BBF, had 5~35% live cells with some dead cells (red coloured) in all compartment group. At 12.5%, there were full of live cells at all compartment groups while the middle layer of ZFF and the bottom layers of VBF and ZFF revealed fewer live cells (~75%) than the control group.

### 3.2. Depth of Cure and Vickers Hardness

**Figure 3:**
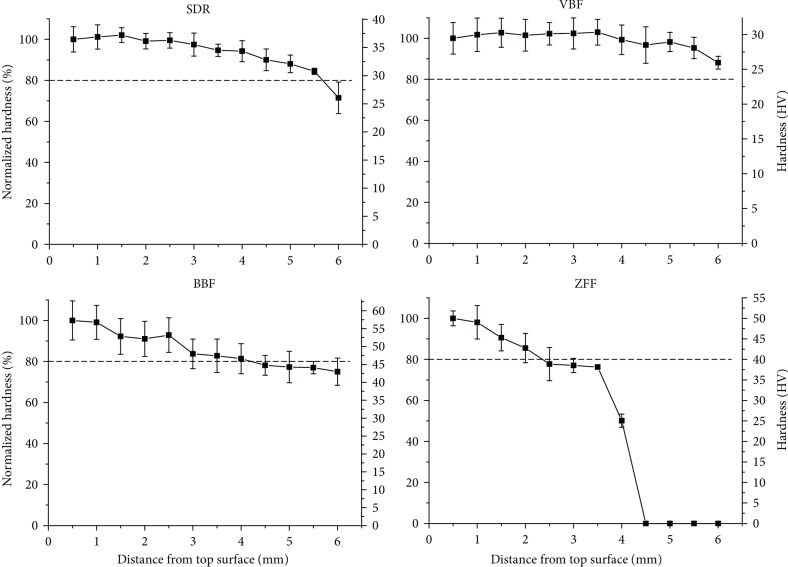
Vickers hardness (*n* = 9) depending on the distance from the top surface of bulk-fill and conventional flowable resins after a single light-curing session. The dotted line indicates 80% normalized hardness compared with the value at 0.5 mm. The bulk-fill resins (SDR, VBF, and BBF) maintained a normalized hardness of approximately 70-90% even at 6 mm from the top surface, while that of the conventional flowable resin (ZFF) decreased to 0% after 4.5 mm.

The measured depths of cure obtained by the scraping test for the bulk-fill composite resins SDR, VBF, and BBF and a conventional flowable resin ZFF were 4.02 mm (±0.11), 3.96 mm (±0.51), 3.55 mm (±0.15), and 2.25 mm (±0.54), respectively (Table 3). According to the Vickers hardness values, depending on the distance from the light-cured top surface to the end of the specimen in 6 mm increments (Figure 3) and the depth of cure determined by the Vickers hardness comparison method, the depths of cure according to the 0.8 ratio of the region of interest/top hardness for the bulk-fill composite resins SDR, VBF, and BBF, as well as the conventional flowable resin, ZFF, were calculated as 5.71, >6 (not determined), 4.24, and 2.35 mm, respectively (Table 3). Overall, a gradual decrease in the normalized Vickers hardness was observed from the top to the bottom surfaces. In detail, SDR, VBF, and BBF reached 71.5, 88.1, and 75.0% hardness at a 6 mm distance, while the conventional flowable resin ZFF reached 50.1% hardness at 4 mm and significantly decreased to 0% hardness after 4.5 mm. A value of 0% was expressed when the Vickers hardness could not be detected in unpolymerized specimens.

### 3.3. Antidifferentiation Effects of Elute

**Figure 4:**
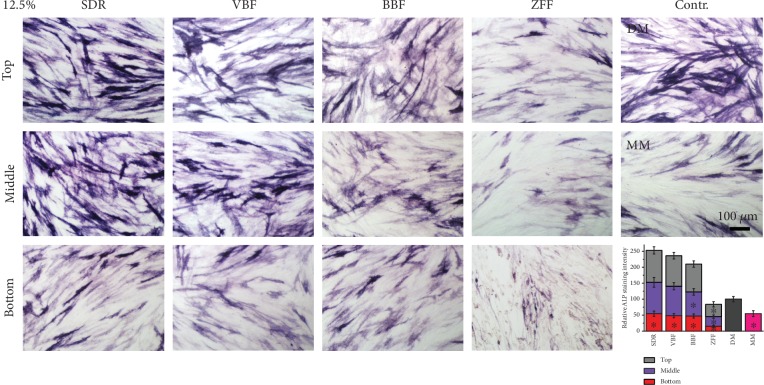
Antidifferentiation effects of elute. Odontogenesis of hDPSCs was evaluated by ALP staining after coculture with 12.5% elute for 7 days. ALP staining intensity was quantified and is shown in the bar graph. Generally, the deeper the specimens extracted for coculture, the less ALP staining observed. ALP staining from all bulk-fill resins was ranked as follows: top ≥ middle > bottom. The flowable resin, ZFF, showed the least amount of ALP staining among the experimental groups (*n* = 5, *p* < 0.05). The asterisks inside the bars indicate significant differences compared with DM (*n* = 5, *p* < 0.05).

To determine any adverse effects of elute from the bulk-fill composite resins, as an early marker of odontogenesis, alkaline phosphatase staining was performed. We chose 12.5% elute, which revealed 75-100% cell viability, to exclude cytotoxicity-induced antidifferentiation effects. Generally, all bottom extractions from bulk-fill resins showed significantly lower ALP staining than the differentiation media control (*p* < 0.05), while all top and middle extractions from the bulk-fill resins showed similar ALP staining (*p* > 0.05), except for the middle extraction from BBF (Figure 4). The deeper specimen was used for gathering extractions for coculture, and less ALP staining was observed. ALP staining from the bulk-fill resins was ranked as follows: top ≥ middle > bottom. The flowable resin, ZFF, exhibited the least amount of ALP staining between the experimental groups (*p* < 0.05).

## 4. Discussion

Mitochondrial enzyme activity-based cell viability assay (WST) and live and dead staining were performed to investigate any compromised cell viability potential from bulk-fill composite resins depending on the distance from light polymerization site. All of the evaluated composite resins except for BBF showed cytocompatibility (~100%) in the top (0-2 mm) of the specimens by WST and live and dead assay. Non-PRG (prereacted glass ionomer) bulk-fill composite resins, such as SDR and VBF, still yielded relatively high cell viability in the middle and bottom compartments (2-4 and 4-6 mm), which is in agreement with previous studies [24, 29, 30]. In contrast, incubation with extracts from a conventional composite resin (ZFF) resulted in greater cytotoxicity by WST and less live cell numbers than that of SDR and VBF, which could be explained by the fact that the depth of cure (by the scraping method) from ZFF (2.25 mm) was much less than that from SDR and VBF (~4 mm). Previous studies also revealed that the depth of cure of ZFF was no greater than 3 mm based on all types of depth of cure tests [31, 32]. The definition of depth of cure is the thickness of resin monomers that may be converted to polymers under light curing, which is dependent on material shade, filler size, monomer composition, light power, and curing time [31]. To equally polymerize resins, the light condition was uniformly set to 1000 mW/cm^2^ for 20 s, which is the commonly recommended intensity and light-curing time according to the manufacturer's instructions. When the thickness of samples for polymerization exceeds the depth of cure, unpolymerized monomers can be released in extracted vehicles and may be more likely to exert cytotoxicity. Accordingly, the samples from the 4-6 mm depth, which exceeded the depth of cure by the ISO 4049 for all four composites, were insufficiently cured, and high amounts of uncured resin monomers were eluted, resulting in cytotoxicity.

Based on another calculation of depth of cure determined by the ratio through serially measured hardness profiles between the top and bottom surfaces and considering the distance with a ratio of 0.8 as the depth of cure based on the hardness profile, the determined depth (2.35~5.71) of cure by hardness profile was not over 4~6 mm except VBF (>6 mm), implicating possible elution of cytotoxic ingredients from the unpolymerized materials. Regarding depth of cure by the ISO 4049 and hardness profiles, there have been many reports implicating overestimation of depth of cure depending on the methodology used for measurements (i.e., scraping test versus hardness profile), specimen size, and light-curing time [33, 34]. In this investigation, to obtain similar conditions between specimens for extraction and depth of cure by the hardness profiles, 4 light-curing sessions (20 s × 4 times) were performed to cover the surface area (*ϕ* = 10 mm) through a relatively small diameter (*ϕ* = 5 mm) of the LED light-curing machine, while a single light-curing session (20 s × 1 time) was implemented for specimen fabrication (*ϕ* = 4 mm) for the scraping test, in accordance with the ISO standard. Generally, the depth of cure obtained by the scraping test was overestimated compared with that obtained by the hardness profile (depth of cure; scarping test > hardness profile) [33]. However, in contrary to other studies, the depth of cure based on the hardness profile was overestimated due to differences in specimen size and light-curing time in this investigation (depth of cure; scarping test < hardness profile). Combining the above results, the bulk-fill resins exhibited greater depths of cure than the conventional flowable resin, supporting the decrease in cytotoxicity observed in fluoride-free bulk-fill resins compared with ZFF.

BBF is a PRG, which is reported to induce cytotoxicity from glass ionomer-based bulk-fill resins, which release greater amounts of fluoride and other cytotoxic ions, such as aluminium, boron, and potassium, which is why BBF showed higher cytotoxicity than the other experimental groups, while the degree of conversion from BBF was not much different from that of other bulk-fill composite resins [23]. Among the released ions from BBF, fluoride ions are regarded to have a major role in cytotoxicity by inhibiting enzyme activity and producing ROS in hDPSCs [35].

The above cell viability results of SDR and ZFF corresponded to those of Toh et al., who reported in vitro cell viability with L929 mouse fibroblasts after exposure to eluates from 2 to 4 mm thick specimens. According to the mitochondria activity assay performed in the previous study, SDR showed the highest cell viability at both 2 and 4 mm, while the standard composite resin ZFF was less cytocompatible at 4 mm. The present study further revealed that bulk-fill resins (SDR and VBF) and ZFF were more cytotoxic to hDPSCs at polymerization depths of 4-6 mm, where the use of bulk-fill resins may be prohibited due to concerns of cytotoxicity to hDPSCs.

Bulk-fill composite resins were introduced to reduce clinical time and effort by a single filling rather than incremental fillings. They are indicated to be used in cavities up to a depth of 4 mm, which is greater than the suggested depth of conventional composite resins (2 mm). Despite the benefits of bulk-fill resins, it is likely that the curing light may not penetrate to the very bottom; thus, resin monomers are left in the nonpolymerized area. Monomers, such as bisphenol A-glycidyl methacrylate (Bis-GMA), triethylene glycol dimethacrylate (TEGDMA), urethane dimethacrylate (UDMA), and (hydroxyethyl)methacrylate (HEMA), are known to be toxic [36, 37]. There are reports of cytotoxic effects on osteoblast-like or dental pulp stem cells from the above monomers [38, 39]. In most clinical cases, bulk-fill resins are used to fill in the cavity, which is prepared down into the dentin. Therefore, uncured monomers or eluates from them in bulk-fill resins can cross the dentinal tubule and eventually affect the cells in the pulp tissue, such as hDPSCs. In this study, possible adverse effects regarding differentiation of hDPSCs were evaluated. The abovementioned uncured or other eluted substances have the possibility to compromise not only cell viability but also other biological activities, including hDPSC differentiation [40]. There was a significantly compromised differentiation of hDPSCs from even the least cytotoxic 12.5% elute from the bottom specimen (4-6 mm), indicating possible adverse effects to pulp tissue from bulk-fill resins located in deep cavities across the dentinal tubules. In this study, hDPSCs were selected because they are one of the most commonly utilized cell types for pulp tissue and they are easy to obtain and use for investigations of in vitro cytocompatibility due to their long viability and active proliferation [9, 17, 41–43]. Furthermore, there are no ethical issues associated with them, as hDPSCs are extracted from third molars, which are human-derived waste. However, investigations of the biological effects against other types of cells in pulp tissue, such as macrophages, neurons, and fibroblasts, are necessary to enhance our knowledge regarding possible adverse effects on pulp tissue from bulk-fill resins in deep cavities [44].

In this study, the cytotoxic effects of three bulk-fill composite resins and a conventional composite resin on hDPSCs were examined according to different polymerization depths of 0-2 (top), 2-4 (middle), and 4-6 (bottom) mm. The 2 mm thicknesses of the cylindrical moulds, which represented the top, middle, and bottom, were stacked to obtain a total thickness of 6 mm, which is close to the maximum length from the occlusal surface of enamel to the roof of the pulp chamber [45]. Polyethylene films were put in between the moulds to facilitate separation of each layer. There may be another way to separate a 6 mm thick resin sample into three compartments (i.e., cutting). However, heat is generated during the cutting process; thus, thermal curing and heat-induced monomer evaporation may occur. Moreover, monomers may be washed out by the water used to cut the samples. Polyethylene film between each compartment (top-middle and middle-bottom) is transparent, and most of the polymerizing light can pass through without mitigation (data not shown), but there is interference resulting from the thickness of the film (1 mm), which was ignored in this study.

Different types of in vitro cytotoxicity tests are proposed in the ISO 10993-5 guidelines [46]. The cells are directly exposed to the dental materials in the direct tests, whereas barriers, such as agar or filters, are placed between cells and materials in the indirect tests to mimic clinical adjustment of materials [47–49]. In particular, when dental materials meet tissue through fluid, elutes of materials in appropriate amounts and types of vehicles (media or distilled water) can be cocultured with cell types of interest to mimic clinical conditions [37, 50, 51]. To assess the various degrees of cytotoxicity derived from bulk-fill resins depending on the depth of cure, this study was performed using extraction due to its sensitivity for quantification [29, 52].

## 5. Conclusion

In conclusion, there was a difference in the cytotoxicity of bulk-fill resins depending on the depth from the light-curing site in the following order: 4-6 mm>2-4 mm>0-2 mm. The depth of cure of various bulk-fill composite resins differed and was greater than that of a conventional, flowable resin. Moreover, elute of specimens from deep cavity regions (4-6 mm) mitigated the differentiation of hDPSCs, necessitating the consideration of bulk-fill composite resin types and light-curing conditions, especially for deep depths of restoration. In addition, certain bulk-fill resins, such as BBF, and conventional composite resins, such as ZFF, should be limited to deeper depths of tooth cavity restorations due to their cytotoxic and antidifferentiation potential.

## Figures and Tables

**Table 1:** Product details of the composite resins used in this study.

Flowable type		Composition	Inorganic fillers	Filler Wt%/Vol%	Curing time	Maximum increment thickness (mm)	Lot number	Manufacturer	
Bulk-fill	SDR	Modified UDMA, bis-EMA, TEGDMA	Barium-alumino-fluoroborosilicate glass, strontium alumino-fluoro-silicate	68%/45%	20 s	4	170302	Dentsply	USA
	Venus bulk-fill (VBF)	UDMA, EBADMA	Ba-Al-F silicate glass, YBF_3_, SiO_2_	65%/38%	20 s	4	10204	Heraeus Kulzer	Germany
	Beautifil Bulk Flowable (BBF)	Bis-GMA, TEGDMA, UDMA, bis-MPEPP	S-PRG filler based on Fluoroboroaluminosilicate glass	73%/not mentioned	10 s LED	4	101721	Shofu Inc.	Japan

Conventional	Filtek Z350 XT flowable restorative (ZFF)	Bis-GMA, TEGDMA, procrylat resins	Ytterbium trifluoride, silica, zirconia/silica cluster	65%/46%	20 s 400-1000 mW/cm^2^, 10 s 100-2000 mW/cm^2^	2	N860177	3M	USA

**Table 2:** hDPSC viability (*n* = 6) after culture in 100% extract from each product.

Product	Top (0~2 mm)	Middle (2~4 mm)	Bottom (4~6 mm)
SDR	99.8 (±9.4)^a,^^∗^	99.2 (±4.8)^a,^^∗^	69.0 (±9.4)^b,^^∗^
VBF	99.1 (±5.6)^a,^^∗^	71.1 (±4.6)^b,&^	62.5 (±4.8)^c,^^∗^
BBF	43.5 (±4.2)^a,&^	7.0 (±2.2)^b,$^	5.5 (±2.9)^b,&^
ZFF	97.1 (±2.3)^a,^^∗^	64.4 (±8.9)^b,&^	5.9 (±4.8)^c,&^

Letters (a, b, and c) represent significant differences between different letters in the same material. Symbols (∗, &, $, and #) represent significant differences between different symbols at the same depths resulting from light curing.

**Table 3:** Depth of cure obtained by the ISO 4049 test and hardness comparison method and summary of cytotoxicity at different depths.

Materials	Cytotoxicity^#^			Depth of cure (mm)^∗^	
	Top (0~2 mm)	Middle (2~4 mm)	Bottom (4~6 mm)	Scraping test (ISO, *d* = 4 mm)	Hardness profile method (region of interest/top = 0.8, *d* = 10 mm)
SDR	-	-	+	4.02 ± 0.11	5.71
VBF	-	+	+	3.96 ± 0.51	>6
BBF	+	+	+	3.55 ± 0.15	4.24
ZFF	-	+	+	2.25 ± 0.54	2.35

^#^When the average cell viability was >70%, noncytotoxicity was determined (-). If the cell viability was <70%, cytotoxicity was determined (+). ^∗^To measure the depth of cure, the scraping test (*n* = 5) used 20 s of light curing, while the hardness profile method (*n* = 9) included 4 sessions of 20 s of light curing to cover a larger diameter.

## Data Availability

The data used to support the findings of this study are included within the article.
